# Purity-Based Continuity Bounds for von Neumann Entropy

**DOI:** 10.1038/s41598-019-50309-7

**Published:** 2019-09-26

**Authors:** Junaid ur Rehman, Hyundong Shin

**Affiliations:** Department of Electronic Engineering, Kyung Hee University, 1732 Deogyeong-daero, Giheung-gu, Yongin-si, Gyeonggi-do 17104 Korea

**Keywords:** Quantum information, Qubits

## Abstract

We propose continuity bounds for the von Neumann entropy of qubits whose difference in purity is bounded. Considering the purity difference of two qubits to capture the notion of distance between them results into bounds which are demonstrably tighter than the trace distance-based existing continuity bounds of quantum states. Continuity bounds can be utilized in bounding the information-theoretic quantities which are generally difficult to compute.

## Introduction

Quantum information science is filled with plethora of intractable problems which range from hard to perform optimizations to finding the optimal state on an infinite dimensional Hilbert space for a given information processing task^1–5^. One prime example of the first kind of problems is calculating the Holevo capacity–which is simply the quantum counterpart of classical channel capacity under certain conditions–of an arbitrary quantum channel^6–9^. The second kind of problems includes calculation of unconstrained quantum and classical capacities of quantum channels, which requires optimization over an unbounded number of channel uses^10–13^.

One effective method to attack the difficult problems of the first kind is to perform the optimization over a smaller set of candidate solutions and then bound the difference of the obtained solution from the actual solution^14–16^. This method to manage difficult problems requires certain mathematical tools which guarantee a bounded difference of the obtained solution from the actual solution. The strength of available tools directly affects the quality of results obtained by this strategy. Main tools which are particularly useful in this method are data processing inequalities of different entropic measures, monotonicity of different distance measures under quantum maps, and the continuity bounds of different information and entropy based measures.

Due to the significance of these tools and their important role in improving *several* important results *at once*, many researchers have worked on these fundamental tools of information theory and have produced numerous results to showcase, e.g., a sharp estimate of von Neumann entropy was proposed^17^ which relied on its continuity. Similarly, uniform continuity bounds on the conditional and relative entropy were also proposed^18^. Recently a bouquet of such continuity bounds was presented^19^ which resulted in some interesting and unexpected majorization relations. These continuity bounds can be effectively utilized to obtain useful bounds on the entropic quantities such as quantum conditional mutual information, Holevo quantity, and various capacities of quantum channels^14^.

In this work we show that the trace distance-based continuity bounds on the von Neumann entropy can be arbitrarily loose. Then, we derive new intuitive continuity bounds which are based on the difference between the purity of two states. This approach gives us continuity bounds which are significantly tighter than the aforementioned bounds.

The remainder of this paper is organized as follows. We begin the Results section with a motivating example for obtaining tight continuity bounds on fundamental quantities like quantum entropy. Then, we review the existing trace distance-based continuity bounds on von Neumann entropy. We show that these bounds can be arbitrarily loose. We present purity-based new continuity bounds on the von Neumann entropy of qubits. In Discussion section, we provide numerical and analytical examples which demonstrate the tightness of prutiy-based continuity bounds of qubits. Finally, we provide the derivation of main result of this paper in the Methods section.

## Results

In this section we present a motivating example for bounding the optimal performance of a fictitious information-theoretic protocol by finding a near-optimal input state. We do not specify the protocol that we are optimizing to stress the generality of the aforementioned method of attacking the hard optimization problems over the Hilbert space.

Let us assume that we are assigned the task of finding the optimal performance characterized by a scalar figure-of-merit *C* over a certain type of quantum channel ${\mathscr{N}}$. We are told that the $C({\mathscr{N}}\,)$ is given as

1 $$C({\mathscr{N}}\,)=\mathop{{\rm{\max }}}\limits_{\rho \in  {\mathcal B} ( {\mathcal H} )}f({\mathscr{N}}(\rho ))$$

where $f({\mathscr{N}}(\rho ))$ is some entropic formula, ${\mathcal B} ( {\mathcal H} )$ is the set of all density operators, and ${\mathscr{N}}$ is a quantum channel which acts on *ρ*, i.e., it outputs the state ${\mathscr{N}}(\rho )$ by operating on *ρ*. In our optimization attempt, we find out that $f({\mathscr{N}}(\rho ))$ is not disciplined in *ρ*, i.e., it is either not monotonic in *ρ* or involves some operations which are not disciplined and hinders the gradient-based optimization routines. This leaves us the following two choices.

First, we can utilize heuristics-based methods, e.g., genetic algorithms, particle swarm optimization, and brute force etc. These methods are time consuming, their complexity grows exponentially with the increase in the dimensionality of the involved Hilbert space, no information on the optimality of the returned solution can be guaranteed, and there does not exist a quantifiable tradeoff between the quality of returned solution and the consumed time in these heuristics-based methods.

**Figure 1:**
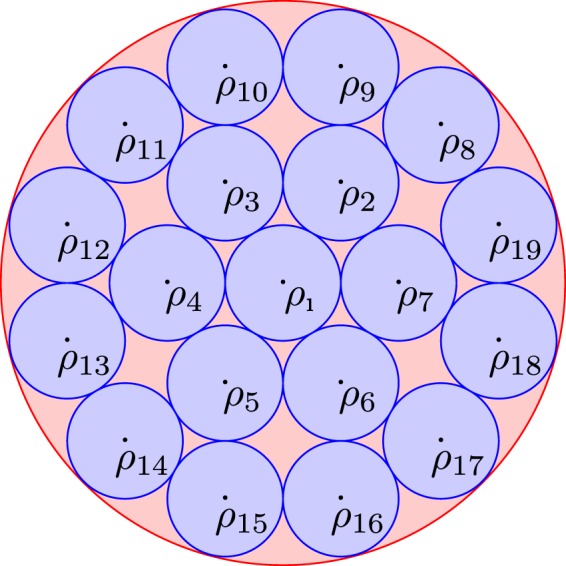
Choosing a finite set of states for evaluation over a continuum of states makes an intractable problem easier to handle.

Second, we invoke the method discussed at the beginning of this article, i.e., we intelligently choose a smaller set of quantum states and perform optimization on this set alone. This method is not only less resource and time intensive than the heuristics-based methods, but we can also guarantee the deviation of obtained solution from the actual solution. We begin by intelligently sampling the Hilbert space, e.g., as shown in Fig. 1 where we have chosen 19 quantum states to form a set ${{\mathscr{C}}}_{19}$ such that for any arbitrary quantum state *ρ*, there exists a *ρ*_*i*_ such that the distance between *ρ* and *ρ*_*i*_, *D*(*ρ*, *ρ*_*i*_), is bounded by *ε*, i.e. for *any* fixed $\rho \in  {\mathcal B} ( {\mathcal H} )$, we have

2 $$\mathop{{\rm{\min }}}\limits_{{\rho }_{i}\in {{\mathscr{C}}}_{19}}D(\rho ,{\rho }_{i})\le \varepsilon .$$

Then, we optimize (1) on ${{\mathscr{C}}}_{19}$ as

3 $$\tilde{C}({\mathscr{N}}\,)=\mathop{{\rm{\max }}}\limits_{{\rho }_{i}\in {{\mathscr{C}}}_{19}}f({\mathscr{N}}({\rho }_{i})).$$

Next we bound the distance between ${\mathscr{N}}({\rho }_{i})$ and that of the optimal output state ${\mathscr{N}}(\rho )$ by using the contractivity of the distance measure *D*(·, ·) under trace-preserving completely positive map ${\mathscr{N}}$, i.e.,

4 $$\varepsilon \ge D(\rho ,{\rho }_{i})\ge D({\mathscr{N}}(\rho ),{\mathscr{N}}({\rho }_{i})).$$

Finally, we invoke the distance *D*(·, ·)-based continuity bounds on *f*(·) of the form

5 $$D(\rho ,{\rho }_{i})\le \varepsilon \Rightarrow |\,f({\mathscr{N}}(\rho ))-f({\mathscr{N}}({\rho }_{i}))|\le g(\varepsilon ),$$

where *g*(·) is some function capturing the continuity of the measure *f*(·). From (1–5), we have

6 $$C({\mathscr{N}}\,)\le \tilde{C}({\mathscr{N}}\,)+g(\varepsilon ),$$

i.e., we have performed the optimization on a set of finite cardinality where it is guaranteed that the optimal performance $C({\mathscr{N}})$ does not deviate by more than *g*(*ε*) from the performance obtained on a reduced set of candidate solutions. Furthermore, one can easily tweak the set ${{\mathscr{C}}}_{19}$ to make *ε* as small as one pleases to obtain a solution which is very close to the actual solution.

It is clear from the above example that the choice of distance measure *D*(·, ·), the quality of set of candidate solutions, and the strength of continuity bounds *g*(*ε*) dictate the quality of simplified optimization on the smaller set of intelligently chosen states. In the next subsection we discuss the trace distance-based continuity bounds on the von Neumann entropy of quantum states.

### Continuity bounds with trace distance

Trace distance is one natural distance measure induced by the trace norm on the space of density operators. Operationally, it gives a measure of distinguishability between quantum states. For two quantum states *ρ* and *σ* on a finite dimensional Hilbert space *H*, the trace distance is defined as

7 $$T(\rho ,\sigma )\equiv \frac{1}{2}||\rho -\sigma |{|}_{1},$$

where $||A|{|}_{1}={\rm{t}}{\rm{r}}(\sqrt{{A}^{\dagger }A})$ is the trace norm (Schatten *p*-norm with *p* = 1). It is a bonafide distance measure, i.e, it is symmetric, nonnegative, and satisfies the triangle inequality. Furthermore, it is contractive under quantum maps, i.e., for two states *ρ* and *σ*, and some quantum channel ${\mathscr{N}}$, we have

8 $$T(\rho ,\sigma )\ge T({\mathscr{N}}(\rho ),{\mathscr{N}}(\sigma )).$$

Note that, *T*(*ρ*, *σ*) = 1 if the states *ρ* and *σ* are orthogonal (their supports are orthogonal), and *T*(*ρ*, *σ*) = 0 iff *ρ* = *σ*.

The von Neumann entropy *S*(·), 0 ≤ *S*(·) ≤ log*d* of *d*-dimensional quantum states (we use log with base 2)

9 $$S(\rho )=-{\rm{tr}}(\rho \,\log \,\rho )$$

is the quantum counterpart of Shannon entropy of probability distributions. It is the most fundamental entropic quantity in the quantum information theory and is used in the definition of several other quantum information-theoretic quantities, e.g., quantum mutual information, Holevo information of quantum channels, coherent information of quantum channels etc. Due to its fundamental nature and utility in the definition of other entropic quantities, any result concerning the von Neumann entropy affects several quantities of interest in the field of quantum information theory. Note that the von Neumann entropy captures the notion of mixedness of quantum states, i.e., *S*(*ρ*) = 0 iff *ρ* is pure, and *S*(*ρ*) = log*d* iff *ρ* is the maximally mixed state on a *d*-dimensional Hilbert space.

One interesting feature of von Neumann entropy is its continuity on the finite dimensional quantum systems. This fact is beautifully captured by the continuity bounds which bound the difference in the von Neumann entropies of states which are close according to some distance measure. The most common distance measure used in these continuity bounds is the trace distance. One fundamental Lemma concerning the continuity of von Neumann entropy in trace distance of quantum states is as follows.

#### Lemma 1

*For states ρ and σ* on a Hilbert space of dimension *d* < *∞*, if $T(\rho ,\sigma )\le \varepsilon \le 1$, then^17,18^

10 $$|S(\rho )-S(\sigma )|\le (\begin{array}{ll}\varepsilon \,\log (d-1)+h(\varepsilon )\, & {\rm{if}}\,\varepsilon \le 1-\frac{1}{d},\\ \log (d)\, & {\rm{if}}\,\varepsilon  > 1-\frac{1}{d},\end{array}$$

where $h(\varepsilon )=-\varepsilon \,\log (\varepsilon )-(1-\varepsilon )\log (1-\varepsilon )$ is the binary Shannon entropy.

Since the von Neumann entropy is bounded as $0\le S(\rho )\le \,\log (d)$ for any quantum state, the maximum value of the left hand side of above expression is trivially log(*d*). Now we are going to show that there exist quantum states such that the left hand side of above bound is zero but the right hand side computes to log(*d*), thus resulting into a trivial bound with no meaningful information.

Since the trace distance is not a measure of mixedness of quantum states, above bound can be arbitrarily loose. To see this, consider that *ρ* and *σ* are both pure and orthogonal to each other, i.e.,

11 $$\rho =|{\psi }_{\rho }\rangle \langle {\psi }_{\rho }|,\,{\rm{and}}\,\sigma =|{\psi }_{\sigma }\rangle \langle {\psi }_{\sigma }|,$$

12 $$\langle {\psi }_{\rho }|{\psi }_{\sigma }\rangle =0.$$

**Figure 2:**
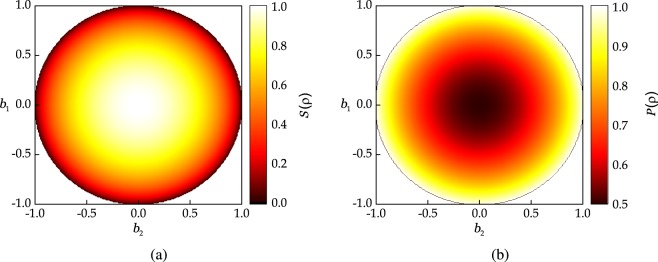
(**a**) Entropy, and (**b**) purity of all states lying on the circle obtained by fixing *φ* = 0. Note that fixing the distance from the center of the circle defines both the purity and the entropy of resulting set of states.

**Figure 3:**
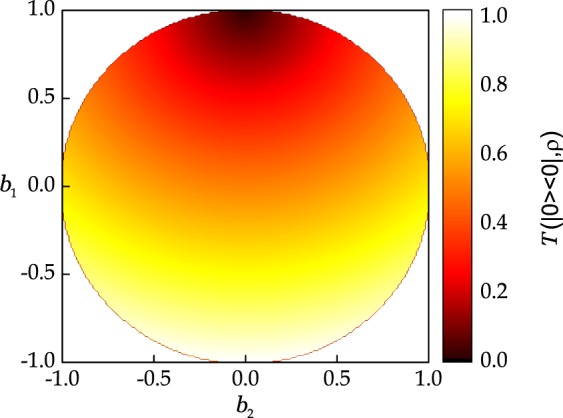
Trace distance of all qubit states lying on the circle obtained by fixing *φ* = 0 from the state |0〉.

Since the considered states are pure, $|S(\rho )-S(\sigma )|=0$, but the right hand side of the above bound is log(*d*) because $T(\rho ,\sigma )=1$. This example can be better visualized on a 2-dimensional Hilbert space. Any qubit state *ρ* can be represented on the Bloch sphere where the states at the antipodal points of the sphere are orthogonal to each other. Let us fix *φ* = 0, so that we have a circle with top as $|0\rangle$ and bottom as $|1\rangle$. The von Neumann entropy of all states lying on this circle is shown in Fig. 2(a). Let us choose $\rho =|0\rangle \langle 0|$, then the trace distance of all states in the circle with *φ* = 0 is plotted in Fig. 3. We can readily verify that choosing $\rho =|0\rangle \langle 0|$ and $\sigma =|1\rangle \langle 1|$ produces the deviation of log*d* = 1, thus showing the arbitrary deviation of the bound (10) for *d* = 2.

### Purity-based continuity bounds

In this section, we choose the difference between the purity of two states as a measure of their closeness, then we bound the difference in their entropies based on this measure. The main motivation of using the difference in the purity as our measure of closeness of quantum states is that both entropy and the purity characterize the mixedness of a quantum state. This qualitative statement can be well understood by comparing the entropy in Fig. 2(a) and the purity in Fig. 2(b). Since both of them are constant for all states lying on the circle of a given radius in the Bloch sphere, continuity bounds based on purity can yield better results than the trace distance-based continuity bounds.

The purity $P(\cdot ),1/d\le P(\cdot )\le 1$ of a quantum state is defined as

13 $$P(\rho )={\rm{tr}}({\rho }^{2}).$$

It attains the minimum and the maximum value for the maximally mixed and pure quantum states, respectively. Figure 2(b) shows the purity of quantum states lying on the circle by fixing *φ* = 0 in the Bloch sphere. Since the edge of the circle contains the pure states, their purity value is equal to 1. In this section, we will consider the difference of purity of two qubits as a (quasi) distance. However, it is important to note that the difference between the purity of two qubits is not a distance measure in strict sense, as for any two states *ρ* and *σ*

14 $$|P(\rho )-P(\sigma )|=0\nRightarrow \rho =\sigma .$$

However, it is nonnegative, symmetric, and obeys the triangle inequality. Consequently, this quasi-distance measure possesses many desirable properties, e.g., unitarily invariance, contractivity under some channels, e.g., depolarizing channel, and its utility in obtaining continuity bounds as given in this article.

One useful representation of a *d*-dimensional quantum state is its Bloch vector representation which is given by^20^

15 $$\rho =\frac{1}{d}(I+\mathop{\sum }\limits_{i=1}^{{d}^{2}-1}{b}_{i}{\sigma }_{i}),$$

where *σ*_*i*_ along with the identity *I* define a matrix basis of the Hilbert space on which *ρ* is defined, *b*_*i*_ are (generally) real numbers satisfying

16 $$\mathop{\sum }\limits_{i=1}^{{d}^{2}-1}|{b}_{i}|=1.$$

For qubit (*d* = 2) case, this matrix basis can be given by the well-known Pauli operators, i.e.,

$${\sigma }_{1}={\sigma }_{X}=[\begin{array}{ll}0 & 1\\ 1 & 0\end{array}]$$

$${\sigma }_{2}={\sigma }_{Y}=[\begin{array}{cc}0 & -\dot{\iota }\\ \dot{\iota } & 0\end{array}]$$

$${\sigma }_{3}={\sigma }_{Z}=[\begin{array}{ll}1 & 0\\ 0 & -1\end{array}].$$

For a general *d*, the elements of matrix basis satisfy the following properties

- All components, except *I*, of the matrix basis are traceless, i.e., ${\rm{tr}}({\sigma }_{i})=0$, and
- The elements of matrix basis are unitary and pairwise orthogonal, i.e.,

17 $${\rm{tr}}({\sigma }_{i}^{\dagger }{\sigma }_{j})=d\,{\delta }_{ij},$$

where *δ*_*ij*_ is the Kronecker’s delta function.

It is straightforward to verify that the Pauli matrices defined above satisfy these properties. Additionally, they are Hermitian, i.e., ${\sigma }_{i}^{\dagger }={\sigma }_{i}$. Given a density matrix *ρ*, the elements *b*_*i*_ can be calculated by

18 $${b}_{i}={\rm{tr}}(\rho {\sigma }_{i}).$$

It can be seen from the above properties and from (18) that *b*_*i*_ are always real if *σ*_*i*_ are Hermitian. Otherwise, *b*_*i*_ can be complex.

The purity of a state in the Bloch vector representation is

19 $$\begin{array}{rcl}P(\rho ) & = & {\rm{tr}}({\rho }^{2})\\  & = & {\rm{tr}}(\frac{1}{d}(I+\mathop{\sum }\limits_{i=1}^{{d}^{2}-1}{b}_{i}{\sigma }_{i})\frac{1}{d}(I+\mathop{\sum }\limits_{j=1}^{{d}^{2}-1}{b}_{j}{\sigma }_{j}))\\  & = & \frac{1}{d}(1+{\Vert b\Vert }^{2}),\end{array}$$

where $b=({b}_{1},{b}_{2},\cdots ,{b}_{{d}^{2}-1})$ is the Bloch vector, and $\Vert \cdot \Vert$ is the vector norm.

For a qubit state *ρ* with the Bloch vector *b* and the eigenvalues *λ*_1_ and *λ*_2_, the following relation holds^21^

20 $${\lambda }_{1}{\lambda }_{2}=\frac{1-{\Vert b\Vert }^{2}}{4}.$$

#### Theorem 1

*Let ρ and σ* be two qubit states such that

21 $$|P(\rho )-P(\sigma )|\le \varepsilon .$$

Then,

22 $$|S(\rho )-S(\sigma )|\le h(\sqrt{\varepsilon /2}).$$

*Proof*. See Methods section.

## Discussion

**Figure 4:**
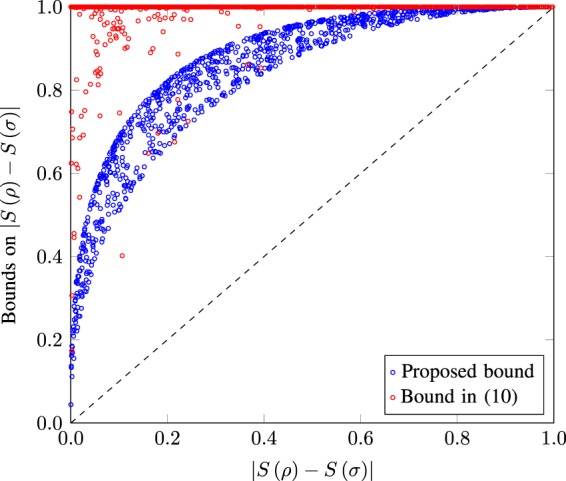
Visual representation of the bound of (10) and that of (22) for 1,000 randomly generated pairs of *ρ* and *σ*. Dashed line shows the actual value of the entropy difference.

Now we present numerical examples comparing the (10) and (22). Figure 4 depicts the numerical evaluation of two bounds ((10) and (22)) for randomly generated pairs of quantum states. Circles lying closer to the diagonal dashed lines indicate a tighter bound. It can clearly be seen that the proposed bound (22) is in general tighter than the existing bound of (10). It is also clear from Fig. 4 that not only the proposed bound gives better results than the trace distance-based continuity bounds, but its deviation from the actual values is also bounded.

**Figure 5:**
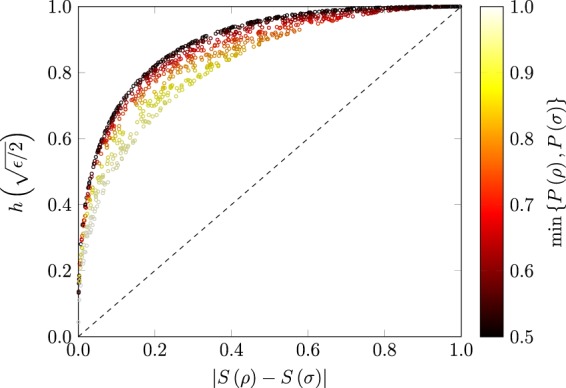
Proposed bound on 1,000 randomly generated qubit states. The deviation of the proposed bound is affected by the location of states on the Bloch sphere.

Figure 5 shows the proposed bound on the pairs of qubit states where the color of the marks indicates the smaller of the two purities of quantum states. It can be seen from Fig. 5 that the maximum deviation of the proposed bound is obtained when one of the states is near to the maximally mixed state (purity ≈ 0.5). Similarly, the inner side of the trend seen in Fig. 5 is composed of states having comparatively higher purity values. These trends clearly indicate that as the inner of the two states moves closer to the edge of the Bloch sphere, the deviation in the bound is reduced.

**Figure 6:**
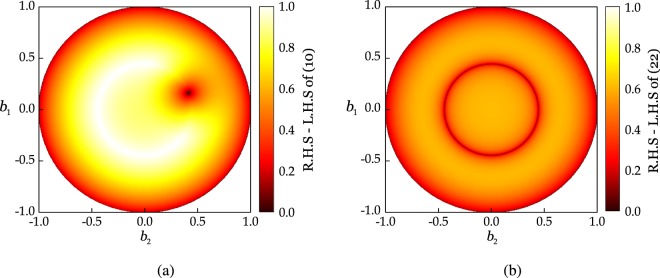
Comparison of (10) and (22) when *ρ* is fixed on the circle with *φ* = 0. (**a**) The difference of left hand side (L.H.S) from the right hand side (R.H.S) of (**a**) trace distance-based continuity bound of (10), and (**b**) purity-based continuity bounds of (22).

The closeness condition (21) ensures that the difference in the distance of both states from the center of the Bloch sphere is bounded. Since the purity does not depend on the exact location on the Bloch sphere, but only on the distance of the state from the center, fixing one state *ρ* at an arbitrary location and then rotating *σ* over the whole circle of a fixed radius will result into the same value of the bound (22). Thus, we can produce a whole set of states, lying on the same distance from the center as *ρ* that will have *S*(*σ*) = *S*(*ρ*). On the other hand, (10) relies on the trace distance. Since the trace distance between two states is zero if and only if both states are the same. Hence, (10) does not possess the same quality of producing a whole set of states whose exact entropy can be predicted. Specifically, it gives the condition *S*(*σ*) = *S*(*ρ*) if and only if *ρ* = *σ*. This different behavior of (22) and (10) is depicted in Fig. 6 where *ρ* is fixed (prominent in Fig. 6(a) by the black point in the interior of the circle) and *σ* are cycled over whole circle with *φ* = 0. Figure 6 shows the difference of left hand side from the right hand side and it numerically confirms the behavior described above.

Continuity bounds on entropic quantities play an important role in finding useful bounds on the performance of several information-theoretic protocols. Von Neumann entropy is the most fundamental quantity and several other quantities in the quantum information theory are based on the von Neumann entropy. We have proposed continuity bounds on the von Neumann entropy of qubits where we have measured the closeness between them by calculating the difference between their purities. This approach results into continuity bounds that are significantly tighter than the best known existing results.

While it is tempting to consider generalizing these bounds to arbitrary *d* case, it turns out to be a challenging task. Obtaining a general expression similar to (20) for the eigenvalues of a general *d*-dimensional quantum state is a challenge. This generalization becomes further complicated when one realizes that the general geometrical structure (e.g., in terms of Bloch vectors) of quantum states is not well understood beyond *d* = 2 case. While efforts are being put into understanding the general geometric structure of higher dimensional Hilbert spaces, the progress seems to be slow and challenging.

One interesting and useful future direction is to consider the unconventional measures of distance, similar to one that we considered in this paper, to find useful continuity bounds on the entropic quantities that are less fundamental in nature, e.g., coherent information, quantum mutual information, and the Holevo information.

## Methods

### Proof of theorem 1

Since

23 $$|P(\rho )-P(\sigma )|\le \varepsilon ,$$

therefore, by using (19), we have

24 $$|{\Vert {b}_{\rho }\Vert }^{2}-{\Vert {b}_{\sigma }\Vert }^{2}|\le 2\varepsilon ,$$

where *b*_*ρ*_ and *b*_*σ*_ are Bloch vectors of *ρ* and *σ*, respectively. Now, without a loss of generality we assume that ${\Vert {b}_{\rho }\Vert }^{2}\ge {\Vert {b}_{\sigma }\Vert }^{2}$, hence,

25 $${\Vert {b}_{\rho }\Vert }^{2}\le {\Vert {b}_{\sigma }\Vert }^{2}+2\varepsilon .$$

From (20), we have

26 $${\lambda }_{1}^{\rho }{\lambda }_{2}^{\rho }=\frac{1-{\Vert {b}_{\rho }\Vert }^{2}}{4}=x,$$

27 $${\lambda }_{1}^{\sigma }{\lambda }_{2}^{\sigma }=\frac{1-{\Vert {b}_{\sigma }\Vert }^{2}}{4}=y,$$

where ${\lambda }_{i}^{\rho }$ and ${\lambda }_{i}^{\sigma }$ are the eigenvalues of *ρ* and *σ*, respectively. Using (24) in the above expressions, we obtain

28 $$x=y-\varepsilon /2.$$

Since the sum of eigenvalues is unity, we write (26) as

29 $${\lambda }^{\rho }(1-{\lambda }^{\rho })=x.$$

Similarly, from (27) we have

30 $${\lambda }^{\sigma }(1-{\lambda }^{\sigma })=y,$$

which are quadratic equations that we can easily solve and obtain

31 $${\lambda }^{\rho }=\frac{1\pm \sqrt{1-4x}}{2}.$$

It is easy to see that the eigenvalue with the addition of the square root term in the above expressions is larger than 1/2 and is clearly the larger of the two eigenvalues, therefore we can bound the difference between the largest eigenvalues of *ρ* and *σ* as

32 $$\begin{array}{rcl}{\lambda }_{{\rm{\max }}}^{\rho } & = & \frac{1+\sqrt{1-4x}}{2}\\  & = & \frac{1+\sqrt{1-4y+2\varepsilon }}{2}\\  & \le  & \frac{1+(\sqrt{1-4y}+\sqrt{2\varepsilon })}{2}\\  & = & {\lambda }_{{\rm{\max }}}^{\sigma }+\sqrt{\varepsilon /2}.\end{array}$$

Hence,

33 $$|{\lambda }_{{\rm{\max }}}^{\rho }-{\lambda }_{{\rm{\max }}}^{\sigma }|\le \sqrt{\varepsilon /2}.$$

Therefore, the difference between the entropy is bounded as

34 $$\begin{array}{rcl}|S(\rho )-S(\sigma )| & \le  & |h({\lambda }_{{\rm{\max }}}^{\rho })-h({\lambda }_{{\rm{\max }}}^{\rho }+\sqrt{\varepsilon /2})|\\  & \le  & h(\sqrt{\varepsilon /2}),\end{array}$$

where the last inequality follows from the fact that ${\lambda }_{{\rm{\max }}}^{\rho }\ge 0.5$ and the binary Shannon entropy, *h*(*x*), is a monotonically decreasing function for 0.5 ≤ *x* ≤ 1.
